# Impact of social capital, sex and education on the utilization of public health services: a cross sectional study based on the China migrant dynamic survey

**DOI:** 10.1186/s12889-021-10803-y

**Published:** 2021-04-19

**Authors:** Zhen Yang, Cheng-hua Jiang

**Affiliations:** 1 School of Medicine, Tongji University, No 1239 Siping Road, Yangpu District, Shanghai, 200092 China; 2 School of Medicine, Jinggangshan University, Jianan, 343009 Jiangxi China

**Keywords:** Social capital, Sex, Education, Public health Services,Moderating effect, Migrants

## Abstract

**Background:**

China is making efforts to promote the equalization of National Essential Public Health Services (NEPHS) for internal migrants. Studies have demonstrated that the impacts of social capital on health services are different among subgroups of people. Clarifying these differences will help China accurately promote the equalization of NEPHS for the internal migrants and provide reference for other countries.

**Methods:**

Data from the China Migrant Dynamic Survey of 2017, involving 130,642 migrants in 31 provinces were used to clarify the complex relationship between social capital and the utilization of NEPHS. Social capital was divided into regional cognitive social capital (RCSC), regional structural social capital (RSSC), individual cognitive social capital (ICSC), and individual structural social capital (ISSC). Then, multi-level logistic regression was conducted to analyze their impacts on the utilization of NEPHS of the migrants, and whether such impacts are moderated by sex and education.

**Results:**

(1) There are significant differences in the levels of CSC, SSC, and NEPHS utilization between different sexs and educational subgroups of the migrants, among which the educational difference is more prominent. (2) An interaction exists between the levels and dimensions of social capital and NEPHS projects. Also, the impact of SSC on NEPHS is always greater than that of CSC at the same level. (3) The effects of RCSC, RSSC, ICSC, and ISSC on NEPHS utilization by migrants are not moderated by sex. However, a high education could weaken the relationship between RCSC and health education, ISSC and health education, and RSSC and health records but strengthen the correlation between RSSC and health education.

**Conclusion:**

Social capital plays an important role in the access of migrants to NEPHS. Governments should vigorously promote the construction of regional social capital, encourage migrants to actively participate in community activities, especially pay attention to the enhancement of the migrants with low SES to the destination identity.

## Background

China is undergoing unprecedented social change. An increasing number of people leave their original places of residence to work and live in other provinces or cities to improve their lives. Such people are called internal migrants. According to the National Health Commission, the Chinese migrant population exceeded 240 million in 2017 [1]. In 2009, China initiated the National Essential Public Health Services (NEPHS) project [2]. It is the NEPHS that the government provides free of charge to all residents in view of the main health problems existing in urban and rural residents, with children, pregnant women, the elderly and patients with chronic diseases as the key groups. Due to the restrictions of household registration system, the migrants is at a disadvantage in accessing NEPHS in their destinations [3, 4]. In 2013, the central government reintroduced measures to promote NEPHS equalization of internal migrants, and since then, NEPHS deployment level has risen rapidly [5, 6]. However, there is still a significant gap in the national planning target [3, 4, 7]. Thus, evaluation of the factors affecting the continuous improvement of the NEPHS utilization by internal migrants and their mechanism of action has become a significant concern that needs immediate resolution.

Social capital refers to the resources and benefits received through connections with others, either as individuals or groups [8]. Social capital is an important social determinant of health, and access to health services has been suggested as a pathway by which Social capital influences health outcomes [9]. The pathways in which social capital influences service accessibility are as follows: (1) promoting the sharing of information among neighbors; (2) changing health behaviors, attitudes, and concepts through interaction with peers [10–12]. Migration means a loss of the original social network and a disruption in civic participation in the new environment [13], which results in the deficiency of the localized social capital of the migrants. Compared with the local population, the social capital of migrants in the destination areas also shows a considerable deficit [14, 15] and is associated with various adverse health outcomes [16–22].

Social capital can be divided into different dimensions (cognitive and structural) and different levels (community and individual). Different types of Social capital affect health services utilization by influencing the availability of health services in communities, the availability and effectiveness of outreach resources between health-care providers and communities they serve, and care-seeking behavior of individuals in those communities [23]. However, Uphoff et al. [24] pointed out that not everyone has access to the same sources of social capital and not everyone will benefit in the same way, they proposed three paths by which Socioeconomic status (SES) could affect the relationship between social capital and health outcomes. (1) A more significant social capital benefit on the health of disadvantaged persons in the society, and no effects or limited health benefits for those in positions higher up in the social ladder. (2) People with a low SES will generally have less social capital, and the capital available to them cannot be used effectively for health benefits. (3) Social capital might benefit the better-off in society while excluding people with a lower SES or a minority position.

In some high income countries, the lack of social capital is reported to significantly limit the effective utilization of local health services by migrant populations [10, 11]. However, due to the interdependence of cultural, economic, and social capital [25], the relationship between social capital and health is not consistent across countries [26, 27]. Notably, the social capital of Chinese people has unique characteristics, with a higher level of trust and a lower level of social participation [28]. The association between social capital and health also presents substantial uniqueness in China [22, 29]. Therefore, it is necessary to continually explore the correlation between social capital and health in the migrant population. However, these previous studies focused more on the health outcomes and neglected the process by which migrants access NEPHS in their destination regions. The study by Hou et al. [30] was an exception, they confirmed the positive effect of individual structural social capital on the health education acceptance and health record establishment of the migrant population.

There are currently at least three unclear dimensions on the relationship between social capital and NEPHS utilization among the Chinese migrants that need further exploration: (1) The influence of different dimensions of individual social capital, both cognitive and structural, on the health outcomes [8]. Although the effect of the latter (structural social capital) on NEPHS utilization has been verified [30], the effect of the former is unclear. (2) The effects of different levels of social capital, both individual and contextual, on the health outcomes [8]. Most studies [22, 30] on the relationship between social capital and NEPHS utilization by migrants were conducted at the individual level, and the discussion was deficient at the contextual level. (3) Differences in the application of social capital and NEPHS by the different migrant population subgroups. The differences in the relationship between social capital and health among different subgroups have attracted increased research attention and could constitute the future focus of social capital [31]. The differences in this relationship between different subgroups of the Chinese migrant population also need to be explored.

In the context of China, a huge low-middle income country (LMIC), this study takes the internal migrants as the respondents to answer three questions: (1) What are the distribution characteristics of individual social capital and NEPHS utilization level among migrant population subgroups with different sex and education levels? (2) How do different dimensions (cognition and structure) and different levels (individual and contextual) of social capital affect the utilization of NEPHS? (3) Do sex and education significantly moderate the relationship between social capital and migrant population NEPHS utilization? The answers to three questions will help us understand the mechanism of social capital on the NEPHS utilization by internal migrants. This will not only help the Chinese government to better promote relevant work, but also provide a meaningful reference for other countries, especially LMICs, to promote the equalization of public health services for the migrants.

## Methods

### Data

The data was obtained from the China Migrant Dynamic Survey (CMDS) in 2017 and was provided by the Migrant Population Service Center. CMDS is an annual national sample survey of the internal migrants organized by the NHC, with an annual sample size of approximately 200,000 households. The survey adopts the layered, multi-stage, and proportional to scale PPS (Probability proportional to size) sampling method. This study adopted the individual questionnaire A, which was uniformly printed and distributed by the NHC. The questionnaire includes basic information about respondents’ demography, perception of the destination, the state of social interaction, and utilization status of NEPHS, etc. Full-time investigators collected the questionnaire data through household interviews, and each respondent gave informed consent before commencing the interview. Dates were entered through the migrant population health and family planning dynamic monitoring system and were checked by the investigators and the investigation instructors. The respondents consisted of internal migrants aged 15–59 living in the destination for more than 1 month. In this study, the inclusion conditions were set as “22-59 years of age, residence duration more than one year, and 1-16 years of education”. Finally, 130,642 people were included.

### Measurement

#### Dependent variables

Health education and health records, the two primary services of NEPHS, were selected as outcome variables. The health education question was “Have you received the following health education in your current community in the past year: occupational disease prevention and control, tuberculosis prevention and control, chronic disease prevention and control, STD and AIDS prevention and control, tobacco control, reproductive health and contraception, maternal and child health care, healthy birth and childbearing, self-help education in public emergencies, and mental health education”. Individuals who had not received any of the above education categories were marked as “No”, while those who had received one or more of the education categories were marked as “Yes”. The health record question was “Have you ever set up a health record at the destination?” and the answer was “yes or no.”

#### Independent variables

Social Capital refers to the social network resources that can be utilized by individuals within the scope of their current residence. It can be distinguished into Cognitive Social Capital (CSC) and Structural Social Capital (SSC). CSC generally refers to individuals’ perceptions, beliefs, and attitudes toward their social surroundings, with corresponding measures focused mainly on the concepts of generalized and particularized trust [32]. The latter was selected in this study, mainly referring to the overall perception of the destination by migrants. There were four questions in the survey: “I like the city/place I live now”, “I am concerned about the changes in the city/place I live now”, “I am very willing to blend with the local people and become a part of them”, “I think the local people are willing to accept me as a part of them”, each question was graded as “totally disagree”, “disagree”, “basically agree”, or “totally agree”, α = 0.844. CSC was divided into four levels: level 1 (4–11), level 2 (12), level 3 (13–15), and level 4 (16).

SSC refers to the presence of formal opportunity structures or activities in which individuals build or strengthen their social connections. These structures and activities are often operationalized through measures of an individual’s civic or social participation [33], and so does this study. The civic participation questions were: “since 2016, have you made suggestions to your unit/community/village or supervised the unit/community/village affairs management”, “since 2016, have you participated in property donation, blood donation, volunteer activities, etc.”, “since 2016, have you reported the situation/put forward policy suggestions to relevant government departments in various ways “, “since 2016, have you posted online comments on national affairs and social events or participated in related discussions”, “since 2016, have you participated in party/youth league organization activities and party branch meetings”. There were four-level answers for each question: no, occasionally, sometimes, and often. The social participation question was “have you participated in any of the following activities in the past year: trade unions, volunteer associations, homecoming associations, fellow-students association, others”. According to the distribution characteristics of scores, civic participation was integrated into two categories: “none” or “at least one”. Social participation was also treated according to this method. According to statistical evaluation, there was a significant correlation between civic participation and social participation (*r* = 0.304, *p* = 0.000). Therefore, the sum of the two was determined as the SSC level, and the three levels were evaluated as either 0, 1, or 2.

#### Moderating variables and controlling variables

Sex and education are often indicators of SES, and SES can significantly influence social capital and health [24]. sex and education were set as moderating variables, According to the compulsory education years in China, education were divided into two categories: ≤9 and > 9 years groups. Several factors, including age, residence duration, migratory range, and community type, have been confirmed to affect the NEPHS utilization of migrant populations in previous studies [3–7]. Thus, the above variables were set as the control variable. The age groups were divided into 22–27, 28–37, 38–47, and 48–59 years old, while the residence time groups were divided into 1–3, 4–6, 7–9, 10–12, and above 12 years. The community types were divided into urban and rural areas, while the migratory range was divided into across provinces, across cities within a province, and across counties within a city.

### Statistical analysis

SPSS 22.0 was used for data analysis. First, the descriptive statistics of the included variables were calculated (Table 1). Then, we compared the differences in social capital, health education, and health records among the different sex and education groups of migrants by cross-table and chi-square tests (Table 2). Our data-set contained a sample of 130,642 individuals nested within 31 provincial administrative units. We calculated the CSC and SSC average grades of the samples from 31 provincial administrative regions, respectively, as the regional cognitive social Capital (RCSC) and regional structural social capital (RSSC) of each region. Then, to distinguish the impact of social capital at different levels on NEPHS, we specified the following basic model:

$$ {\mathit{\mathsf{H}}}_{\mathit{\mathsf{ij}}}={\mathit{\mathsf{\beta}}}_{\mathit{\mathsf{0}}}+{\mathit{\mathsf{\beta}}}_{\mathit{\mathsf{1}}}\left({\mathit{\mathsf{S}}}_{\mathit{\mathsf{ij}}}-{\mathit{\mathsf{S}}}_{\mathit{\mathsf{j}}}\right)+{\mathit{\mathsf{\beta}}}_{\mathit{\mathsf{2}}}{\mathit{\mathsf{S}}}_{\mathit{\mathsf{j}}}+{\mathit{\mathsf{\beta}}}_{\mathit{\mathsf{3}}}{\mathit{\mathsf{X}}}_{\mathit{\mathsf{ij}}}+{\mathsf{\mu}}_{\mathsf{j}}+{\mathit{\mathsf{\varepsilon}}}_{\mathit{\mathsf{ij}}} $$

**Table 1:** Respondent’s Socio-demographic Characteristics in 2017, China (*N* = 130,642)

Variables	Subgroups	N	%
Sex	Male	68,510	52.4
	Female	62,132	47.6
Education	≤9 years	78,491	60.1
	> 9 years	52,151	39.9
Age (years)	22–27	28,348	21.7
	28–37	51,446	39.4
	38–47	35,711	27.3
	47–59	15,137	11.6
Residence duration (years)	1–3	36,034	27.6
	4–6	34,056	26.1
	7–9	23,162	17.7
	10–12	13,968	10.7
	> 12	23,422	17.9
Community type	Urban	97,276	74.5
	Rural	33,366	25.5
Migration range	Across provinces	63,521	48.6
	Across cities within the province	43,512	33.3
	Across counties within a city	23,609	18.1
CSC	Level 1	11,778	9.0
	Level 2	48,024	36.8
	Level 3	36,882	28.2
	Level 4	33,958	26.0
SSC	Level 1	49,554	37.9
	Level 2	45,051	34.5
	Level 3	36,037	27.6
Health education	No	33,711	25.8
	Yes	96,931	74.2
Health records	No	90,927	69.6
	Yes	39,715	30.4

**Table 2:** A cross-table showing the effect of Sex and education on social capital and NEPHS utilization

Variables	Subgroups	Sex differences			Education differences		
		Male	Female	*Χ*^*2*^	1–9 years	10–16 years	*Χ*^*2*^
CSC	Level 1	9.2	8.8	13.847**	10.7	6.5	1006.563***
	Level 2	36.9	36.6		37.7	35.4	
	Level 3	27.9	28.6		27.8	28.8	
	Level 4	26.0	26.0		23.8	29.3	
SSC	Level 1	35.0	41.2	700.850***	35,436	27.1	6677.397***
	Level 2	34.7	34.2		27,265	34.1	
	Level 3	30.2	24.6		15,790	38.8	
Health Education	No	26.9	24.6	93.718***	27.5	23.3	288.741***
	Yes	73.1	75.4		72.5	76.7	
Health Record	No	70.9	68.1	119.316***	70.8	67.8	136.467***
	Yes	29.1	31.9		29.2	32.2	

Note: ***p < 0.001, **p < 0.01,*p < 0.05

where H is the relevant dependent variable for an individual I (level 1) in province j (level 2), S is the set of social capital variables measured at the individual and province levels, X is a vector of standard socioeconomic-demographic variables (log of sex, education, age, residence duration, community type, and migration range). The β’s are the fixed parameters to be estimated, μ_j_ is the province-specific random effect, whereas ε_ij_ is the random component of the error term. Therefore, (S_ij_ - S_j_) refers to the pure personal social capital, which can be divided into individual cognitive social capital (ICSC) and individual structural social capital (ISSC). Thus, the social capital of the migrant population was disassembled into RCSC, RSSC, ICSC, and ISSC. Finally, we added the interaction terms of RCSC, RSSC, ICSC, and ISSC with sex and education into the model to analyze the moderating effect of sex and education on the correlation between social capital, health education, and health records (Tables 3, 4 and 5).

**Table 3:** Logistic regression results of Sex, education, control variables on health education and health records

Independent Variables	Reference group	Baseline model of health education			Baseline model of health records		
		OR	95% CI		OR	95% CI	
Sex							
Male	Female	0.910***	0.887	0.933	0.888***	0.867	0.909
Education							
> 9 years	≤9 years	1.130***	1.099	1.162	1.103***	1.075	1.131
Age (years)							
28–37	22-27 years	1.166***	1.127	1.207	1.071***	1.037	1.106
38–47		1.069**	1.028	1.111	1.091***	1.051	1.132
47–59		0.794***	0.758	0.832	1.036	0.989	1.085
Residence duration							
4–6 years	1–3 years	1.061**	1.025	1.099	1.093***	1.058	1.129
7–9 years		1.017	0.978	1.057	1.069***	1.031	1.109
10–12 years		0.931***	0.890	0.974	1.026	0.982	1.072
> 12 years		0.898***	0.864	0.934	0.973	0.936	1.010
Community type							
Urban	Rural	1.276***	1.241	1.313	1.215***	1.181	1.250
Migration range							
Across cities within province	Across provinces	1.416***	1.376	1.457	1.484***	1.445	1.525
Across counties within a city		1.388***	1.340	1.438	1.568***	1.519	1.620
Cox & Snell R^2^		0.015			0.014		

Note: ***p < 0.001, **p < 0.01, *p < 0.05

**Table 4:** Logistic regression results of social capital on health education

Independent Variables	Model 1a			Model 2a			Model 3a		
	OR	95% CI		OR	95% CI		OR	95% CI	
Sex	0.826***	0.805	0.848	0.830***	0.808	0.852	1.219	0.865	1.717
Education	0.886***	0.860	0.911	0.868***	0.843	0.894	1.463*	1.019	2.101
CSC	1.157***	1.141	1.171						
SSC	2.072***	2.035	2.109						
RCSC				0.834***	0.782	0.890	0.981	0.883	1.091
RSSC				30.809***	27.825	34.114	29.245***	24.737	34.573
ICSC				1.167***	1.151	1.184	1.190***	1.162	1.218
ISSC				1.961***	1.926	1.997	1.987***	1.917	2.042
Sex*RCSC							0.899	0.791	1.021
Sex*RSSC							0.894	0.729	1.096
Sex*ICSC							0.983	0.956	1.012
Sex*ISSC							1.016	0.980	1.053
Education*RCSC							0.742***	0.649	0.848
Education*RSSC							1.382***	1.118	1.710
Education*ICSC							0.972	0.944	1.001
Education*ISSC							0.961*	0.927	0.997
Cox & Snell R^2^	0.072			0.092			0.093		

Note: the regression results controlled all variables in three models as presented in Table 3. ***p < 0.001, **p < 0.01,*p < 0.05

**Table 5:** Logistic regression results of social capital on health records

Independent Variables	Model 1b			Model 2b			Model 3b		
	OR	95% CI		OR	95% CI		OR	95% CI	
Sex	0.845***	0.825	0.866	0.851	0.831***	0.873	0.875	0.619	1.235
Education	0.945***	0.920	0.970	0.932	0.908***	0.958	2.149***	1.507	3.064
CSC	1.231***	1.215	1.247						
SSC	1.457***	1.434	1.480						
RCSC				1.640***	1.542	1.744	1.877***	1.698	2.074
RSSC				9.705***	8.820	10.679	9.464***	8.101	11.056
ICSC				1.214***	1.197	1.230	1.223***	1.197	1.250
ISSC				1.398***	1.376	1.420	1.386***	1.350	1.423
Sex*RCSC							0.948	0.840	1.070
Sex*RSSC							1.137	0.940	1.375
Sex*ICSC							0.996	0.970	1.023
Sex*ISSC							1.009	0.979	1.041
Education*RCSC							0.764***	0.675	0.864
Education*RSSC							0.897	0.738	1.091
Education*ICSC							0.985	0.958	1.012
Education*ISSC							1.009	0.977	1.041
Cox & Snell R^2^	0.041			0.054			0.054		

Note: the regression results controlled all variables in three models as presented in Table 3. ***p < 0.001, **p < 0.01, *p < 0.05

## Results

### Descriptive statistics

Table 1 shows that 52.4% of the migrant population were male; 60.1% had no more than 9 years of education. Further, 61.6% of the population were under the age of 37 years, 53.7% had lived in the destination for less than 6 years, 74.5% lived in cities, and nearly half (48.6%) had moved across provinces. The CSC of the migrant population was high while the SSC was low. Meanwhile, 91.0% of the migrants had a positive evaluation of the destination, but 37.9% did not participate in any activities in the previous year. The acceptance rate of health education among the migrants (74.2%) was significantly higher than that of health records (30.4%).

### Comparison of social capital and NEPHS utilization among different subgroups

According to Table 2, the CSC level of males was lower than that of females, while the SSC level of males was stronger than that of females. The proportion of males receiving health education was significantly lower than that of females, and the proportion of males who established health records was also significantly lower than that of females. Migrants with higher years of education had higher levels of CSC and SSC, and also had higher rates of health education and health records. In summary, the sex differences in the social capital, health education, and health records of migrant populations were significantly smaller than their differences in education years.

### Effects of social capital on NEPHS utilization

The results in Table 3 show the binary logistics regression analysis conducted using age, residence duration, migratory range, community type, sex, and education as independent variables, and health education and health record as the dependent variables. All control variables had a significant impact on health education and health records. The results also show that sex and education had a significant impact on NEPHS utilization and that women and highly educated migrants were more likely to access NEPHS.

After the introduction of CSC and SSC, the R^2^ of the health education Model 1a (Table 4) was significantly improved compared with that of the baseline model. After social capital was disintegrated from RCSC, RSSC, ICSC, and ISSC, the R^2^ of Model 2a was further significantly increased. However, the introduction of interaction terms did not increase the R^2^ of Model 3a significantly. In Model 3a, the OR value of RCSC was not significant; the OR values of RSSC, ICSC, and ISSC were all significantly greater than 1, the interaction terms of sex and RCSC, RSSC, ICSC, and ISSC were not significant. In the interaction terms of education and social capital, the OR value of education*RSSC was significantly greater than 1, while the OR for interactions education*RCSC and education*ISSC were significantly less than 1.

As shown in Table 5, introducing CSC and SSC significantly improved the Model 1b of health records compared with the R^2^ of the baseline model. After the introduction of RCSC, RSSC, ICSC, and ISSC, the R^2^ of Model 2b increased again, whereas introducing the interaction terms did not increase the R^2^ of Model 3b significantly. In Model 3b, the OR values of RCSC, RSSC, ICSC, and ISSC were all significantly greater than 1, and the interaction terms of sex, education and RCSC, RSSC, ICSC, ISSC were only education *RCSC significant and less than 1.

Combining the results shown in Tables 4 and 5, we find that: (1) At both individual and collective levels, CSC always has less impact on utilization of NEPHS than SSC; (2) In comparison, SSC has a more prominent impact on health education, while CSC has a greater impact on health records; (3) The relationship between social capital and health education or health records was only significantly moderated by education. In conclusion, a complex dependency relationship between social capital dimensions, levels, and NEPHS was observed. The relationship between the different dimensions was moderated by education, not sex.

## Discussion

There were three main findings. (1) There are significant differences in the levels of CSC, SSC, and NEPHS utilization between different sexs and educational subgroups of the migrants, among which differences in education years are more prominent. (2) An Interaction exists between the levels and dimensions of social capital and NEPHS projects; the effect of SSC on the NEPHS is always greater than that of CSC at the same level. (3) The effects of RCSC, RSSC, ICSC, and ISSC on NEPHS utilization of migrants are not moderated by sex. However, a higher education could weaken the relationship between RCSC and health education, strengthen the link between RSSC and health education, weaken the relationship between ISSC and health education, and also weaken the association between RSSC and health records.

As some studies have pointed out, the social capital of China’s migrant population is insufficient [14, 15]. However, according to the data in this study, this deficiency may only exist in SSC, and the level of CSC of the Chinese migrant population is still high. Lack of economic and cultural capital bars societal subgroups from acquiring and using social capital [25]. People with high SES have advantages in the acquisition of social capital [24], which is also true for Chinese migrants [19, 34], a fact also confirmed in this study from the gap of social capital in education. A Canadian survey found that men’s CSC was lower while the SSC was higher than that of women [22] . In China, a survey of migrants in Wuhan, Hubei province, did not find sex differences in social capital [19]. Another survey in Shaoxing, Zhejiang province, also found no significant sex difference in social trust and social participation [34]. The sample and different operational definition of social capital could account for the inconsistency. CSC can be divided into generalized and particularized trust [32], the Canadian and Hubei studies used generalized trust, while the Zhejiang study investigated particularized trust (trust to local people). For SSC, it is difficult to compare different studies due to the large differences in the included contents. The concept of CSC and SSC in this study is more similar to that in the Zhejiang, we found that the differences of sex in CSC and SSC, while statistically significant, were small. Which needs to be verified in future studies under a unified concept. As for the sex and education differences in health education and health records, the conclusions of this study are similar to previous studies [3, 5, 6, 35, 36].

Poortinga [26] analysed data from 22 European countries and concluded that individual-level rather than context-level (states) social capital is associated with self-rated health. A similar study in rural China found that CSC, at both individual and contextual (village) levels, is positively correlated with health. Meanwhile, SSC has very low statistical association at both levels [28]. These two studies [26, 28] concur on the ICSC, similar to this study. This study demonstrated that ISSC is positively related to NEPHS, which is also consistent with similar studies [30]. According to Palmer et al. [14], the overall level of social participation in rural China is considerably low, which attributed to the flooring effect [28]. Of note, the bulk of the migrant population is farmer, most of who live in cities, and the social participation of cities is higher than that of rural areas [14]. Awareness is a prerequisite to NEPHS access, and lack of awareness is the main obstacle hindering the utilization of NEPHS by migrant population [37]. Besides, the information function of SSC makes service acquisition a more useful resource for NEPHS. It is worth noting that in this study, RSSC is the most prominent factor affecting the NEPHS, and the effect of RCSC on NEPHS is more complicated. As a government project, NEPHS are more affected by government input. Zhang et al. [6] reported that investment to NEPHS varies among different regions of China. Therefore, social capital at contextual (provinces) level may have a more significant impact on NEPHS. The relationship between RSSC and NEPHS reflected this correlation, but RCSC only had a significant impact on health records. This may be because health education only needs pure information exposure, so information (SSC) is more important. The establishment of health records also requires active cooperation, so recognition (CSC) is more important.

Studies based on the western background reveal sex differences in the relationship between social capital and health outcomes [32, 38–40]. However, this study did not observe any sex difference, which could be attributed to the uniqueness of the Chinese social capital but needs further investigation. In the background of this paper, we mentioned that Uphoff et al. [24] proposed three paths by which SES could affect the relationship between social capital and health outcomes. Based on the analysis results of this study, we infer that: the relationship between education, RCSC, ISSC, and health education conformed to path 1, while the relationship between education, RSSC, and health education conformed to path 2. In particular, this study found that the relationship between education, RCSC, and health records did not correspond to either path and appeared as an aggressive version of path 1. We can speculate that the dimensions and levels of social capital, education, and the types of NEPHS projects jointly affect the utilization level of NEPHS by the migrants in the destination. In summary, the data showed that low-education migrants benefit more from RCSC when accessing NEPHS. This suggests that the local government should attach great importance to improving the local identity of the migrant population when promoting the equalization of NEPHS.

Two problems need to be pointed out. Firstly, this study simplifies health education by merging the nine health education items into one and dualizing the options for “yes or no”. This evaluation criterion is based on the premise that each province attaches equal importance to the nine health education items. In fact, different provinces have different concerns about the health of the migrant population [41], which will affect their choice of health education content and the acceptance rate of the same health education program by the migrants also differs significantly among the provinces [6]. Although the simplified criterion is convenient for overall comparison, it may cause bias in results interpretation. Secondly, as mentioned by Palmer et al. [17], the social capital of Chinese people has its own characteristics, and the cultural difference is more prominent in SSC. Although the measurement indicators of social capital in this study are based on the Chinese cultural background, the mechanism of its influence on public health services is universal, so the conclusion of this study still has certain reference value for other countries.

## Conclusions

The social capital of migrants in China and its relationship with NEPHS utilization have distinct characteristics. The CSC of migrants is high while the SSC is low. Although there are significant sex and educational differences in social capital and NEPHS utilization of migrants, their correlation is not moderated by sex. However, a high education can weaken the relationship between RCSC and health education, strengthen the relationship between RSSC and health education, weaken the relationship between ISSC and health education, and also weaken the relationship between RSSC and health records. Social capital plays an important role in the access of migrant population to services. Governments should vigorously promote the construction of regional social capital, encourage migrants to actively participate in community activities, especially pay attention to the enhancement of the migrants with low SES to the destination identity.

## Data Availability

Since the data used in this paper were provided by the Migrant Population Service Center, which is the top agency governing migrant population issues in China, we had to sign a legally binding agreement with the agency that we will not share any original data with any third parties. However, interested researchers can apply for access to the data at http://www.ldrk.org.cn/.

## Abbreviations

AIDS: Acquired Immune Deficiency Syndrome
CSC: Cognitive Social Capital
SSC: Structural Social Capital
NEPHS: National Essential Public Health Services
ICSC: Individual Cognitive Social Capital
ISSC: Individual Structural Social Capital
RCSC: Regional Cognitive Social Capital
RSSC: Regional Structural Social Capital
SES: Socioeconomic Status
STD: Sexually Transmitted Disease
